# Comparative analysis of community composition and network structure between phyllosphere endophytic and epiphytic fungal communities of *Mussaenda pubescens*

**DOI:** 10.1128/spectrum.01019-24

**Published:** 2024-12-03

**Authors:** Deqiang Chen, Juanjuan Yang, Shunfen Wang, Siren Lan, Yonglong Wang, Zhong-Jian Liu, Xin Qian

**Affiliations:** 1 Fujian Colleges and Universities Engineering Research Institute of Conservation and Utilization of Natural Bioresources, College of Forestry, Fujian Agriculture and Forestry University, Fuzhou, China; 2 Key Laboratory of National Forestry and Grassland Administration for Orchid Conservation and Utilization at College of Landscape Architecture, Fujian Agriculture and Forestry University, Fuzhou, China; 3 Fujian Agriculture and Forestry University, Fuzhou, China; 4 Baotou Teachers’ College, Baotou, China; Pennsylvania State University, University Park, Pennsylvania, USA

**Keywords:** epiphytic fungi, endophytic fungi, community composition, fungal diversity, co-occurrence network

## Abstract

The phyllosphere constitutes a critical habitat for microorganisms, exerting profound influences on host vitality, developmental dynamics, reproductive functions, and stress resilience. However, the diversity and network structure of endophytic and epiphytic fungal communities within this microecosystem have not been thoroughly explored. In this investigation, high-throughput sequencing technologies were employed to assess the diversity, community composition, and network structure of endophytic and epiphytic fungal communities associated with *Mussaenda pubescens* across six geographically distinct locations in Southeast China. The results revealed significant differences in community composition and diversity between endophytic and epiphytic fungi, with pronounced geographical variation observed within these phyllosphere fungal communities. Network analysis indicated that epiphytic fungal networks possess enhanced complexity compared with their endophytic counterparts, although the latter exhibit greater network stability. Moreover, stochastic processes were identified as pivotal in shaping the composition of these fungal communities. This research substantially enriches our comprehension of the diversity and organizational mechanisms of phyllosphere fungal communities, providing novel insights into the modalities of species coexistence and the stability of community equilibrium within ecosystems.

**IMPORTANCE:**

This study employs high-throughput sequencing technologies to explore the fungal communities within the phyllosphere of *Mussaenda pubescens* across Southeast China, offering significant insights into plant mycobiome. It demonstrates geographical variations in these fungal communities, with epiphytic fungi exhibiting more complex interaction networks compared with the endophytic fungi. Crucially, the research indicates that stochastic processes play a substantial role in the composition of fungal communities. These findings enhance our comprehension of plant-associated microecosystems and underscore the intricate interplay of randomness in maintaining ecosystem stability and diversity.

## INTRODUCTION

Plant-associated microbes are integral in ensuring host vitality, augmenting growth and fecundity, and fortifying resistance to environmental adversities (1–4). The phyllosphere, recognized as one of Earth’s most microbially abundant environments, displays a remarkable diversity of fungal communities. This includes both epiphytic fungi, which colonize leaf surfaces, and endophytic fungi, which inhabit internal leaf tissues. These fungi are vital for the stability of ecosystem functions and biodiversity (5–7). Consequently, an intricate understanding of the diversity and the mechanisms that underpin the composition of these endophytic and epiphytic fungal communities within the phyllosphere is essential for elucidating their roles in the preservation of biodiversity and community equilibrium.

The colonization of phyllosphere microorganisms requires adaptations to specific microecological niches. Endophytic fungi, which maintain close contact with the internal environment of the host plant, extract nutrients directly from plant tissues (8), while epiphytic fungi engage primarily with the external environment, sourcing nutrients from the atmosphere or through leaf penetration (9). Substantial differences in diversity and community composition between these fungal types have been documented (10, 11). Guo et al. (12) revealed strong niche differentiation shaping microbial communities, resulting in a rapid loss of diversity along the decreasing pH gradient from rhizosphere soil to leaves, with sex influencing microbial assembly differently in each niche. Bernard *et al.* (13) also discovered significant differences in the bacterial community structures of *Hibiscus tiliaceus* various ecological niches. These differences in community composition are primarily attributed to the physiological, chemical, and physical variations of plants in different microenvironments (4, 14). Unique ecological niches can shape the structure of the plant microbiome through interactions among plant species, soil physicochemical properties, and numerous other factors. Consequently, different plant compartments may harbor distinct microbial communities (12, 15).

Geographic location plays a decisive role in the distribution patterns of species diversity (16). Geographic variation is also widely recognized as a crucial factor in shaping phyllosphere fungal communities (17). Langenfeld *et al.* (18) found significant differences in the composition of fungal communities on the leaves of *Cephalotaxus harringtonia* across different geographic locations. Similarly, the distribution of endophytic fungal communities in the leaves of *Metrosideros polymorpha* exhibits a pronounced geographic effect (19). Although the composition of phyllosphere microbial communities has been relatively well-studied, our understanding of the variations in the composition and diversity of phyllosphere endophytic and epiphytic fungal communities across different geographic locations remains limited.

Interactions among microorganisms have profound effects on community formation and function. Thorough exploration of co-occurrence networks is not only crucial for understanding the composition of microbial communities but also provides insights into potential relationships and reveals the spatial distribution of ecological niches. This exploration can identify key taxa that may significantly influence community structure and function, regardless of their abundance (20, 21). Additionally, comparing the topological features of networks can help us gain deeper insights into co-occurrence patterns within communities (22, 23). Therefore, studying the co-occurrence patterns and differences between phyllosphere endophytic and epiphytic fungal networks is of significant value for comprehensively understanding the community assembly of phyllosphere fungi.

Furthermore, niche-based theory posits that community structure is predominantly shaped by deterministic processes, which include biotic interactions (such as competition, predation, symbiosis, and trade-offs) and environmental filtering (such as pH, temperature, and humidity), collectively determining the final composition of the community (24–26). In contrast, neutral theory emphasizes the role of stochastic processes (such as ecological drift and dispersal limitation) in shaping microbial community structure (27, 28). Studies have demonstrated that in natural systems, both stochastic and deterministic processes are critical in shaping the composition of microbial communities (29). However, research on phyllosphere fungi remains limited, with a significant gap in understanding how deterministic and stochastic processes influence their community assembly. This gap hinders a comprehensive understanding of phyllosphere fungal ecology, particularly in relation to their adaptation and interaction with environmental factors across spatial and temporal scales.

*Mussaenda pubescens*, a drought-resistant shrub, thrives within the secondary evergreen broad-leaved forests of southern China (30). *M. pubescens* has been traditionally used in Chinese herbal medicine as an antichloristic and antipyretic agent for treating pharyngitis, acute gastroenteritis, and dysentery. Previous research has extensively explored its reproductive characteristics and population genetics (31). However, there remains a relative paucity of knowledge regarding the distribution patterns and network structures of phyllosphere endophytic and epiphytic fungal communities in this plant species. In this study, leaf samples of *M. pubescens* were collected from different geographic locations and analyzed using high-throughput sequencing technology to investigate the composition, diversity, and network structures of both endophytic and epiphytic fungi. The aims of this study are (1) to investigate potential differences in the composition and diversity of endophytic and epiphytic fungal communities at a regional scale and (2) to reveal the differences in network structures between endophytic and epiphytic fungal communities.

## MATERIALS AND METHODS

### Study site and sample collection

In the spring of 2021, we selected six locations within Fujian Province in Southeast China to collect samples for the study of the population structure of *M. pubescens*. The chosen sites were Changle, Datian, Fuzhou, Gutian, Nanping, and Youxi as showed in Qian *et al*. (32), with significant differences in altitude, latitude, and longitude. Five 10 × 10 m² plots were established at each site, with a minimum distance of 10 m between plots. Within each plot, three mature *M. pubescens* individuals were randomly selected. From each individual, 10 to 15 healthy leaves were collected and immediately placed in sterile polyethylene bags. The bags were labeled and stored in foam boxes with ice packs to maintain sample integrity. All samples were stored at −80°C upon arrival at the laboratory, where they were subsequently subjected to DNA extraction. The GPS coordinates (latitude and longitude) were recorded using a handheld GPS device. These sites support substantial wild populations of *M. pubescens*, each with more than 100 individuals, offering representative samples for study.

To collect epiphytic fungi from the leaf surfaces, we employed the methodology proposed by Yao *et al.* (33). Specifically, 5.0 g of frozen leaves were placed into 50 mL plastic tubes containing sterile, cold TE buffer (10 mM Tris-HCl, 1 mM EDTA, pH 7.5). The samples were then subjected to alternating cycles of ultrasonication for 45 seconds and vortexing for 30 seconds, repeated three times. Subsequently, the leaves were transferred to new tubes, and the suspension was centrifuged at 10,000×*g* for 10 min. After discarding the supernatant, the sediment was resuspended in 5 mL of CTAB extraction buffer (comprising 2% CTAB, 100 mM Tris-HCl, 1.4 M NaCl, 20 mM EDTA, 1.5% PVP, 0.5% 2-mercaptoethanol, pH 8.0) and heated to 65°C. Homogenization was conducted for 30 s at 6.0 m/s using a FastPrep−24 device, adhering to the manufacturer’s guidelines. For extracting endophytic fungi, the leaves underwent surface sterilization following the protocol by Guo *et al.* (34), involving a sequence of immersions: first in 75% ethanol for 1 min, then 3.25% sodium hypochlorite for 3 min, and again in 75% ethanol for 30 s, with three subsequent rinses in sterile distilled water. The treated leaves were then lyophilized in liquid nitrogen, homogenized with a sterile mortar and pestle, and transferred to tubes containing preheated CTAB extraction buffer at 65°C for further processing.

### DNA extraction and high-throughput sequencing

Total DNA was extracted from all collected samples utilizing the FastDNA Spin Kit for Soil (MP Biomedicals, USA). The quality and concentration of the extracted DNA were assessed using a NanoDrop2000 spectrophotometer (Invitrogen, Thermo Fisher Scientific, Waltham, MA, USA). Using a two-step library construction method, the first step is to use DNA as a template and design primers with adapters for PCR. The second step is to use the PCR product from the first step as a template for PCR (35). To amplify the fungal ITS2 region, the PCR amplification was performed using primers FITS7 and ITS4 (36, 37). The PCR reaction mixture, with a total volume of 10 µL, consisted of 50 ng of genomic DNA, 0.3 µL of each primer, 5 µL of KOD FX Neo Buffer, 2 µL of dNTPs (2 mM each), 0.2 µL of KOD FX Neo enzyme, and sufficient double-distilled water (ddH_2_O) to complete the volume. The amplification protocol included an initial denaturation at 95°C for 5 min, followed by 25 cycles of 30 s at 95°C for denaturation, 30 s at 50°C for annealing, and 40 s at 72°C for extension, concluding with a final extension at 72°C for 7 min. The PCR products were purified using Agencourt AMPure XP Beads (Beckman Coulter, Indianapolis, IN, USA) and quantified with a Qubit dsDNA HS Assay Kit and Qubit 4.0 Fluorometer (Invitrogen, Thermo Fisher Scientific, Waltham, MA, USA). Following quantification, the PCR products were pooled at equimolar ratios to construct the final sequencing library, which was subsequently sequenced on the Illumina NovaSeq 6000 platform.

### Bioinformatics analysis

FLASH software (version 1.2.11) was utilized for the assembly of raw sequencing data (minimum overlap length of 10 bp and maximum allowable mismatch rate of 0.2 in overlap regions) to ensure accurate sequence reconstruction (38). Subsequently, Trimmomatic software (version 0.33) was employed to perform quality filtering on the assembled sequences. Parameters included a 50 bp window size, trimming of bases from the 3′ end if the average quality within the window fell below 20, and filtering out tags with lengths less than 75% of the tag length post-quality control, resulting in high-quality clean tags (39). This step was followed by the use of cutadapt 1.9.1 (40) to identify and remove primer sequences, achieving clean reads devoid of primer contamination. Subsequent denoising, merging of paired-end sequences, and removal of chimeric sequences (sequence similarity >80%) were conducted using the dada2 algorithm integrated within the QIIME2 framework (41), resulting in a data set of non-chimeric reads. These sequences were clustered into operational taxonomic units (OTUs) at a 97% similarity threshold using USEARCH software (version 10.0). Singletons and the OTUs with abundance <0.005% were removed during the data cleaning phase to minimize noise and potential errors predominantly derived from sequencing artifacts (42). Representative sequences from these OTUs were taxonomically classified to the species level *via* the Unite database (version 7.2) and the RDP classifier (version 2.2, with an 80% confidence threshold) (43, 44), identifying 1,602 unique OTUs. To enable comparative statistical analysis, sequence data were normalized to address variances in sequence counts across samples. The processed sequencing data have been deposited in the NCBI Sequence Read Archive (SRA), under BioProject ID PRJNA1096448.

### Statistical analysis

All analyses in this study were executed within the R programming environment (version 4.3.0). Utilizing the “vegan” package (45), we calculated the Shannon diversity index to quantify alpha diversity and employed permutational multivariate analysis of variance (PERMANOVA) to explore variations in community structure among the samples. Wilcoxon test was used for comparing epiphytes and endophytes (all samples). Kruskal–Wallis nonparametric test was used to obtain the *P* value of the difference across all groups, and the Dunn’s test is a *post hoc* test for differences between each pair of groups (46, 47). The similarity percentages (SIMPER) analysis, also within the “vegan” package, was utilized to identify key phyllosphere fungal species that significantly contributed to community differences (48). There was no significant difference observed at the phylum level, and the diversity of species was limited. Consequently, we opted to conduct our comparison at a more specific taxonomic level, namely the class level. Robust Aitchison distance metric was used to analyze phyllosphere fungal community diversity, and the results were visually represented through principal coordinates analysis (PCoA) (49).

Employing the “WGCNA” package (50), we constructed a co-occurrence network for phyllosphere fungi based on Spearman correlation to analyze the interaction patterns between endophytic and epiphytic fungal communities. Relationships within this network were considered statistically significant when the correlation coefficient between fungal members exceeded |0.6| (screen out fungal members with low correlation coefficient), and the *P*-value was <0.01. Using the “igraph” package, we calculate the node-level topological properties to understand the network characteristics of endophytic and epiphytic fungi communities, and use Gephi software to visually display the network (51, 52). Following the classification framework proposed by Shi *et al.* (53), we identified key fungal operational taxonomic units (OTUs) according to their within-module connectivity Zi and among-module connectivity Pi values, which delineate their specific roles within the network. Network hubs, representing nodes with extensive connections across the entire network, were defined by Zi >2.5 and Pi >0.62; module hubs, indicating nodes with extensive connections within individual modules, by Zi >2.5 and Pi ≤0.62; connectors, which facilitate interactions between distinct modules, by Zi ≤2.5 and Pi >0.62; and peripheral members, characterized by limited connectivity primarily within their own modules, by Zi <2.5 and Pi <0.62.

Furthermore, to determine the potential importance of stochastic process to community composition, we implemented a neutral community model (NCM) to predict the detection frequency of OTUs based on their relative abundance. In this model, Nm is the estimated value of dispersion between communities. The parameter Nm determines the correlation between the frequency of occurrence and the relative abundance of the region, where n represents the size of meta-community and m represents the mobility. The parameter R^2^ represents the global fitting with the neutral model (54). The 95% CI of all fitting statistics was calculated by using 1,000 bootstrap repetitions (55). To quantify habitat specialization, Levin’s niche breadth of each OTU in all samples was calculated by using the “spaa” R package (56). Constructing phylogenetic trees for phylogenetically-based null model analysis (iCAMP) involves a comprehensive workflow from initial data collection to detailed statistical analysis. The ITS sequences of each fungal taxa are then aligned using tools like multiple sequence comparison by log-expectation (MUSCLE), where parameters such as max iterations and gap open penalties are adjusted based on sequence variability to optimize alignment. For the phylogenetic tree construction, algorithms like maximum likelihood (ML) using RAxML, and Bayesian Inference using MrBayes are employed. This refined tree is integrated with community composition data for the null model analysis in iCAMP. Additionally, the phylogenetic normalized stochasticity ratio (pNST) of phyllosphere epiphytic and endophytic fungi was compared using the Wilcoxon test. Based on the approach proposed by Ning *et al.* (57), we employed the iCAMP to further quantify the relative contributions of different ecological processes. These analyses did not link community composition with environmental factors and successfully addressed many ecological phenomena (57).

We subsequently evaluated phylogenetic turnover employing the abundance-weighted β-mean nearest taxon distance (βMNTD) metric, which measures the average phylogenetic distance between each OTU and its nearest relative among community pairs (58). To accommodate stochastic phylogenetic variations, we established null models incorporating 999 randomizations (βMNTD_null_), thereby simulating phylogenetic turnover devoid of selective forces. The β nearest taxon index (βNTI) was calculated by determining the deviation of the observed βMNTD from the average βMNTD_null_ values (58, 59). Values of |βNTI| > 2 signify that taxa are phylogenetically either more closely or more distantly related than would be anticipated by chance, suggesting the influence of selection on community assembly. Specifically, βNTI values exceeding +2 indicate heterogeneous selection (HeS), while values below −2 denote homogeneous selection (HoS). For communities where selection did not govern β-diversity (|βNTI| ≤ 2), we further evaluated OTU taxonomic turnover to infer the influence of dispersal or ecological drift on community structure. This was achieved by calculating the Raup–Crick metric based on Bray–Curtis dissimilarities (RC_bray_), which compares observed β-diversity against that expected under a random community assembly model (58–60). The RC_bray_ metric ranges from −1 to 1, with a value of 0 indicating no deviation from the null expectation. A threshold of |RC_bray_| > 0.95 signifies a significant departure from random community assembly, with absolute RC_bray_ values ≤ 0.95 indicating community assembly driven solely by ecological drift (DR). Conversely, RC_bray_ values >+0.95 or <−0.95 suggest that community assembly is influenced by dispersal limitation (DL) or homogenizing dispersal (HD), respectively (59, 61, 62). The results of these analyses were effectively visualized using the “ggplot2” package (63), ensuring the data were presented clearly and concisely.

## RESULTS

### Phyllosphere fungal community composition and diversity

**Fig 1:**
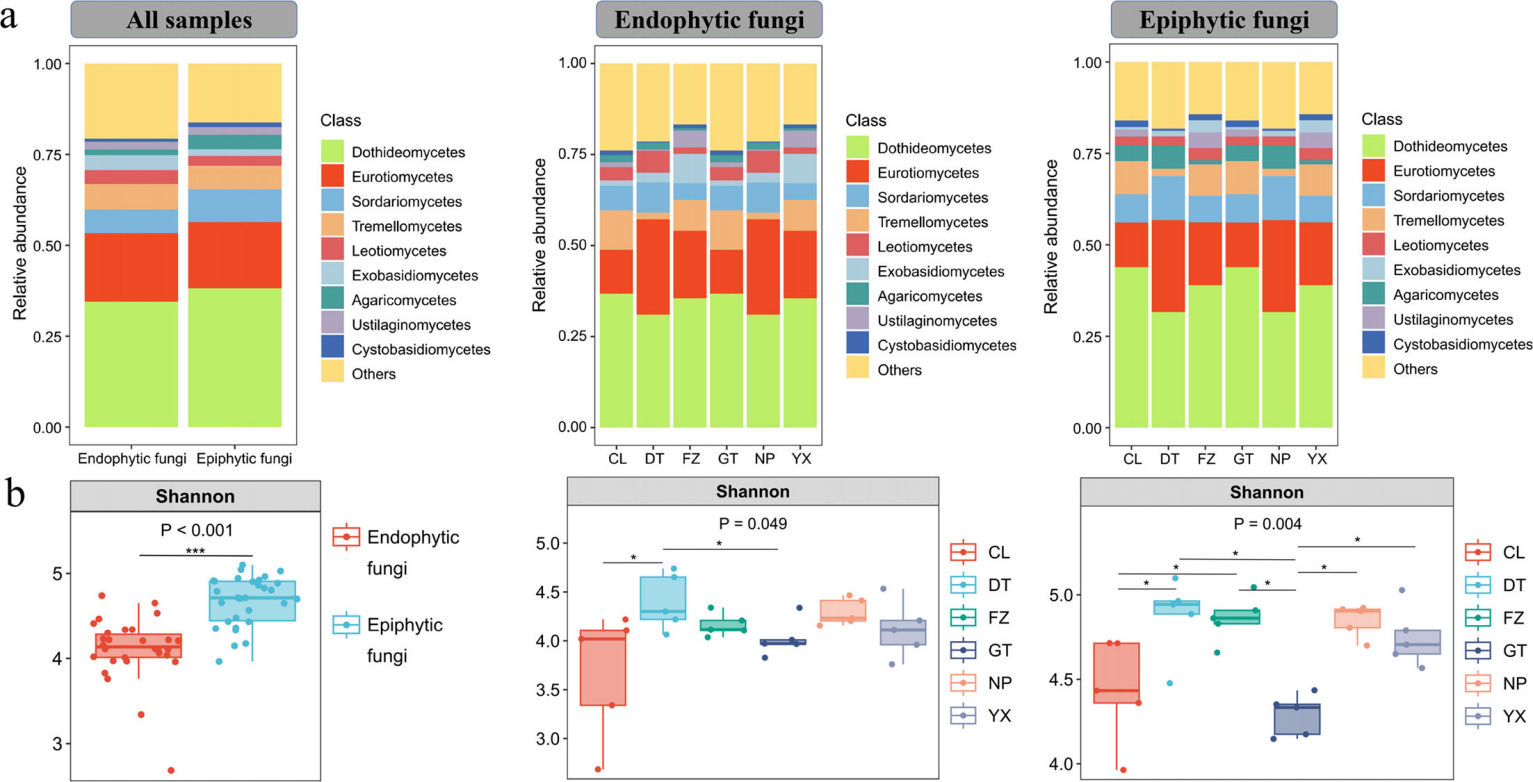
The composition and diversity of phyllosphere fungal communities in *Mussaenda pubescens*. (a) The composition of the *Mussaenda pubescens* phyllosphere fungal community at the class level, based on samples from various geographical locations and ecological niches (endophytic and epiphytic fungi); (b) the alpha diversity (Shannon indices), based on samples from various geographical locations and ecological niches (endophytic and epiphytic fungi). Wilcoxon test was used for comparing epiphytes and endophytes (all samples). Kruskal–Wallis nonparametric test was used to obtain the *P* value of the difference across all groups, and the Dunn’s test is a *post hoc* test for differences between each pair of groups. * indicates significant differences at 0.01 < *P* ≤ 0.05; ** indicates highly significant differences at 0.001 < *P* ≤ 0.01; *** indicates extremely significant differences at *P* ≤ 0.001.

We identified 1,602 operational taxonomic units (OTUs) across all samples through sequencing and analysis of the fungal ITS2 gene. The analysis consistently revealed a dominance of the Dothideomycetes and Eurotiomycetes classes in both endophytic and epiphytic phyllosphere fungal communities, irrespective of geographic origin (Fig. 1a; Fig. S1). Furthermore, the alpha diversity of these fungal communities exhibited significant variations across different geographic and ecological contexts (Fig. 1b; Fig. S2). Specifically, the Shannon diversity of endophytic fungal communities was markedly lower than that of epiphytic communities. Both the alpha diversity of endophytic and epiphytic fungi is highest at the DT site, while it reaches the lowest levels at the CL and GT sites (Fig. 1b). Similarity percentage analysis (SIMPER) highlighted that Eurotiomycetes, Tremellomycetes, and Exobasidiomycetes were principal in shaping the variance in endophytic fungal community composition, whereas Eurotiomycetes, Dothideomycetes, and Tremellomycetes predominantly influenced the epiphytic communities (Tables S1 and S2).

**Fig 2:**
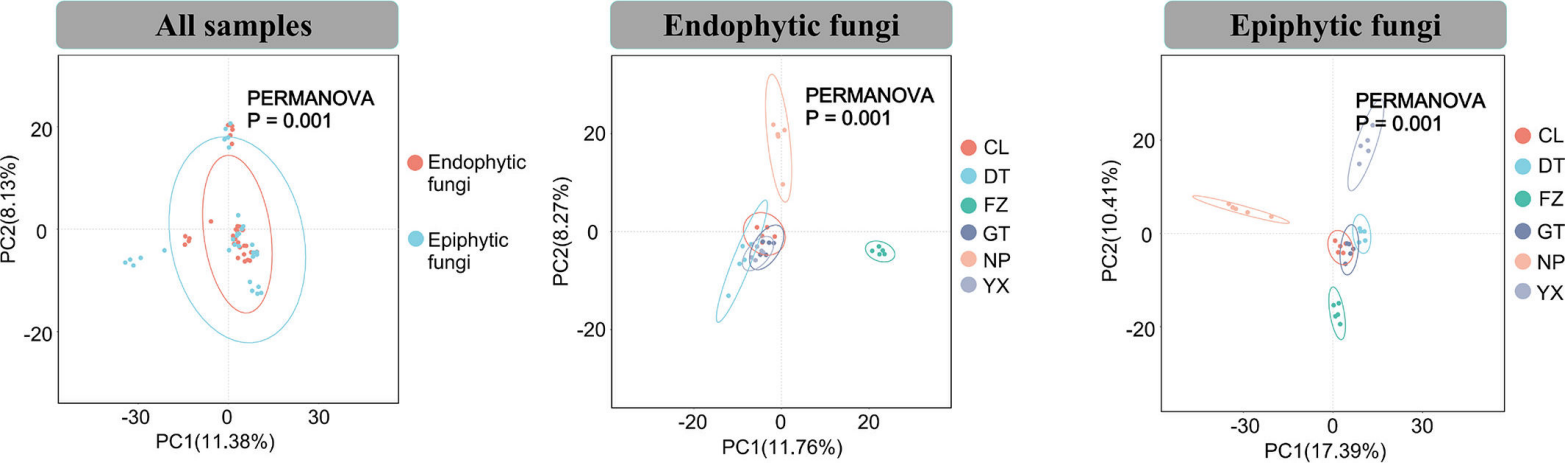
Beta diversity of phyllosphere fungal communities. Principal coordinate analysis (PCoA) and permutational multivariate analysis of variance (PERMANOVA) of phyllosphere fungal communities across various geographical locations and ecological niches.

By employing principal coordinates analysis (PCoA) and permutational multivariate analysis of variance (PERMANOVA) based on robust Aitchison distance, we identified significant differences in the community structure of endophytic and epiphytic fungi. Similarly, substantial differences were observed in the fungal community structures across different geographical locations. These findings indicate that distinct ecological niches and geographical variation significantly influence the composition of phyllosphere fungal communities (Fig. 2; Fig. S3).

### Co-occurrence patterns in phyllosphere fungal communities

**Fig 3:**
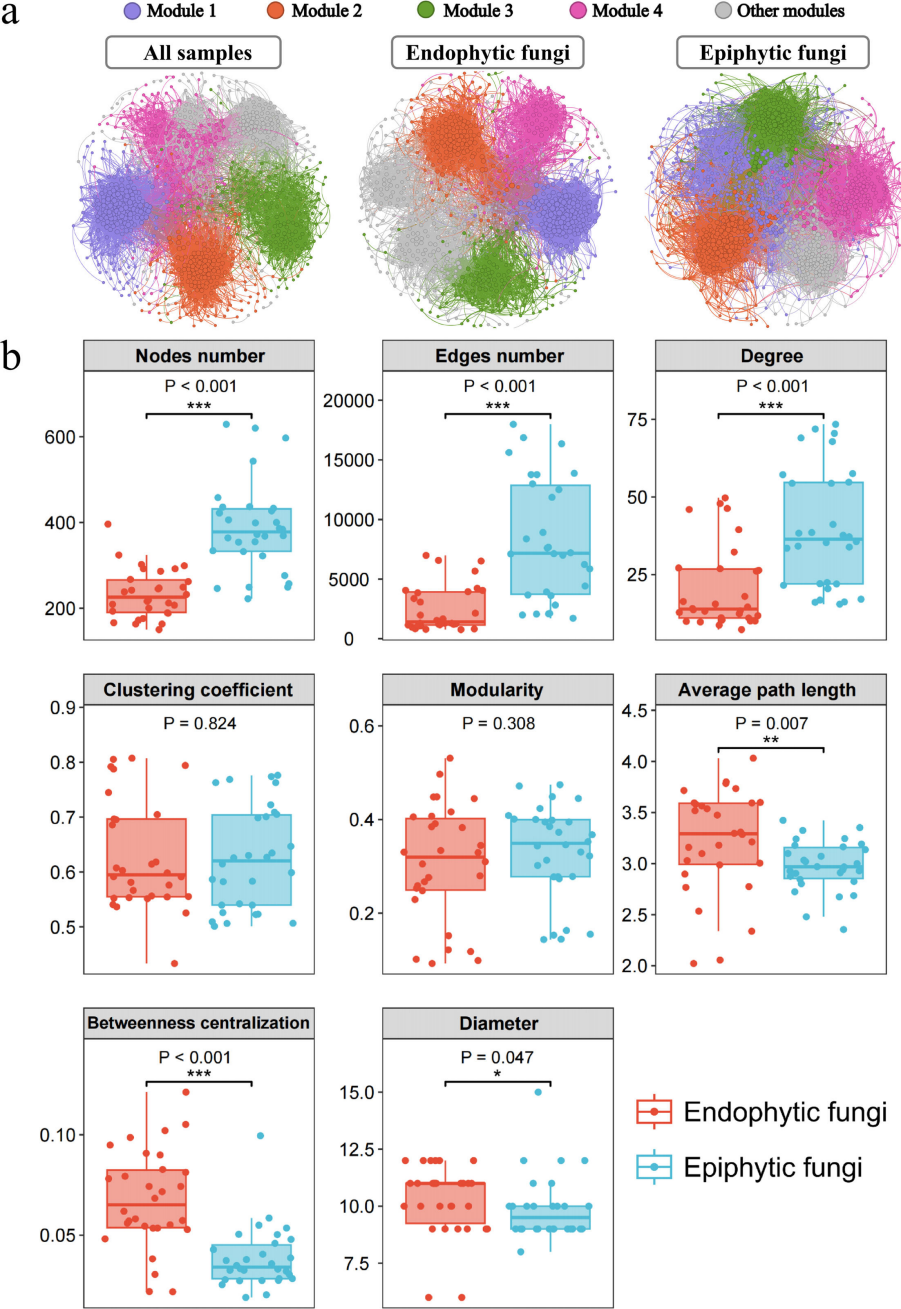
Co-occurrence networks of phyllosphere fungal communities. (a) Network plots show the intra-associations within the all sample, endophytic, and epiphytic fungi sub-communities. Phyllosphere fungal OTUs are represented as nodes and are colored according to network modules. The node size is proportional to the OTU abundances. Lines indicate significant correlations between OTUs, and the line width represents the strength of the correlation coefficient; (b) Network-level topological features of phyllosphere fungal communities (endophytic and epiphytic fungi). Wilcoxon test was used to test the difference between groups. * indicates significant differences at 0.01 < *P* ≤ 0.05; ** indicates highly significant differences at 0.001 < *P* ≤ 0.01; *** indicates extremely significant differences at *P* ≤ 0.001.

Network analysis disclosed pronounced differences between the endophytic and epiphytic fungal networks. Specifically, the endophytic fungal network comprised 839 nodes and 17,075 edges, whereas the epiphytic fungal network contained 1,106 nodes and 44,820 edges, indicative of more complex interactions within the epiphytic communities (Fig. 3a; Table S3). The modularity values of both endophytic and epiphytic fungi were consistently high, all exceeding 0.5, indicating a well-defined modular structure in the constructed networks. Epiphytic fungal networks, in comparison to their endophytic counterparts, demonstrated significantly higher numbers of nodes and edges, as well as enhanced average degrees and network density. These findings indicate a more complex interconnectivity within epiphytic populations (Table S3). Subsequent sub-network analyses further highlighted distinct topological features at the node level, revealing that epiphytic communities possess a more complex and densely connected network structure compared with endophytic fungi (Fig. 3b).

Network nodes, representing phyllosphere fungal OTUs, were categorized into four connectivity-based groups: peripherals, connectors, module hubs, and network hubs. The majority were peripherals, with connections largely restricted to their respective modules. The different subcommunity networks exhibited varying numbers of keystone OTUs. Across the entire network, three module hubs (*Anteaglonium gordoniae*, Capnodiales, Ascomycota) and six connectors (Ascomycota, *Phaeosphaeria podocarpi*, *Hyphozyma roseonigra*, *Eriosporella bambusicola*, Myriangiales, Didymellaceae) were identified. In contrast, within the endophytic network, only one connector (Chaetothyriales) was found, and no keystone OTUs were present in the epiphytic network (Fig. S4). These observations underscore the marked differences in network structure and interaction dynamics between endophytic and epiphytic fungal communities.

### Ecological processes influencing phyllosphere fungal community composition

**Fig 4:**
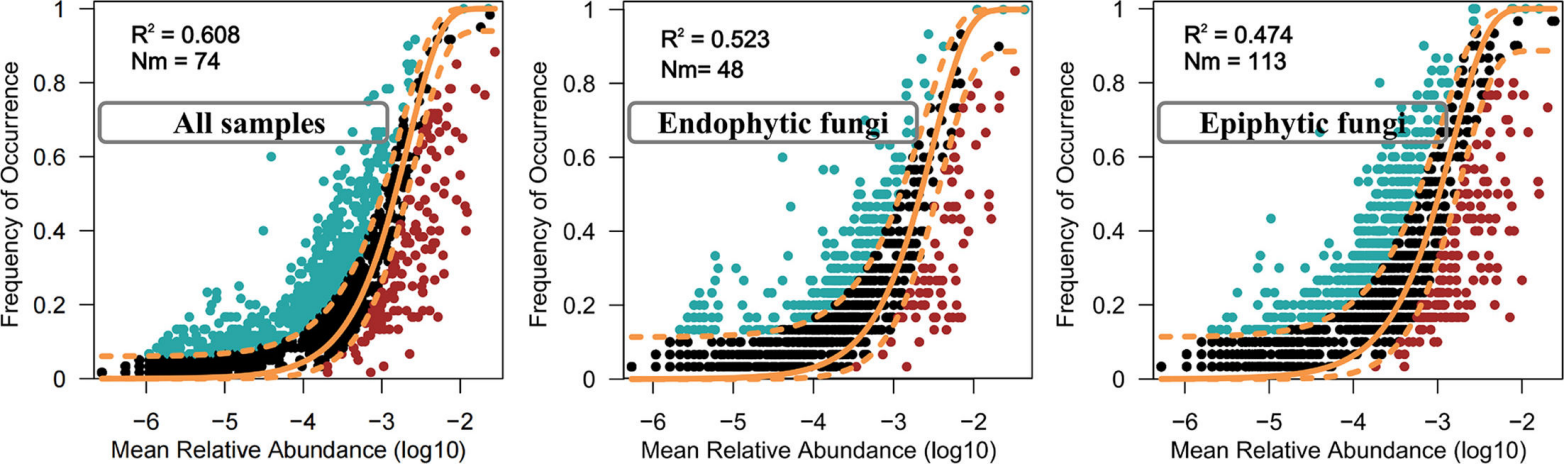
Neutral community models for all sample, endophytic, and epiphytic fungi. The orange solid and dashed lines indicate the predicted occurrence and 95% CI of the neutral model, respectively. R^2^ indicates the goodness of fit to the neutral model, and Nm shows the migration rate.

The neutral model effectively revealed the regularity between the occurrence frequency of fungal OTUs and their relative abundance, providing quantitative evidence for the role of stochastic processes in fungal community composition. Our analysis demonstrates that stochastic processes play a dominant role in shaping the communities of endophytic and epiphytic fungi within the phyllosphere. According to the neutral model, these random processes account for 60.8%, 52.3%, and 47.4% of the community variation in the all samples, endophytic, and epiphytic fungi, respectively. Notably, the dispersal ability of endophytic fungi surpasses that of epiphytic fungi (Fig. 4). Additionally, it was found that the migration rate (m value) of epiphytic fungi was significantly higher than that of endophytic fungi. Moreover, niche width analysis revealed that endophytic fungi occupy a narrower ecological niche compared with epiphytic fungi (Fig. S5).

**Fig 5:**
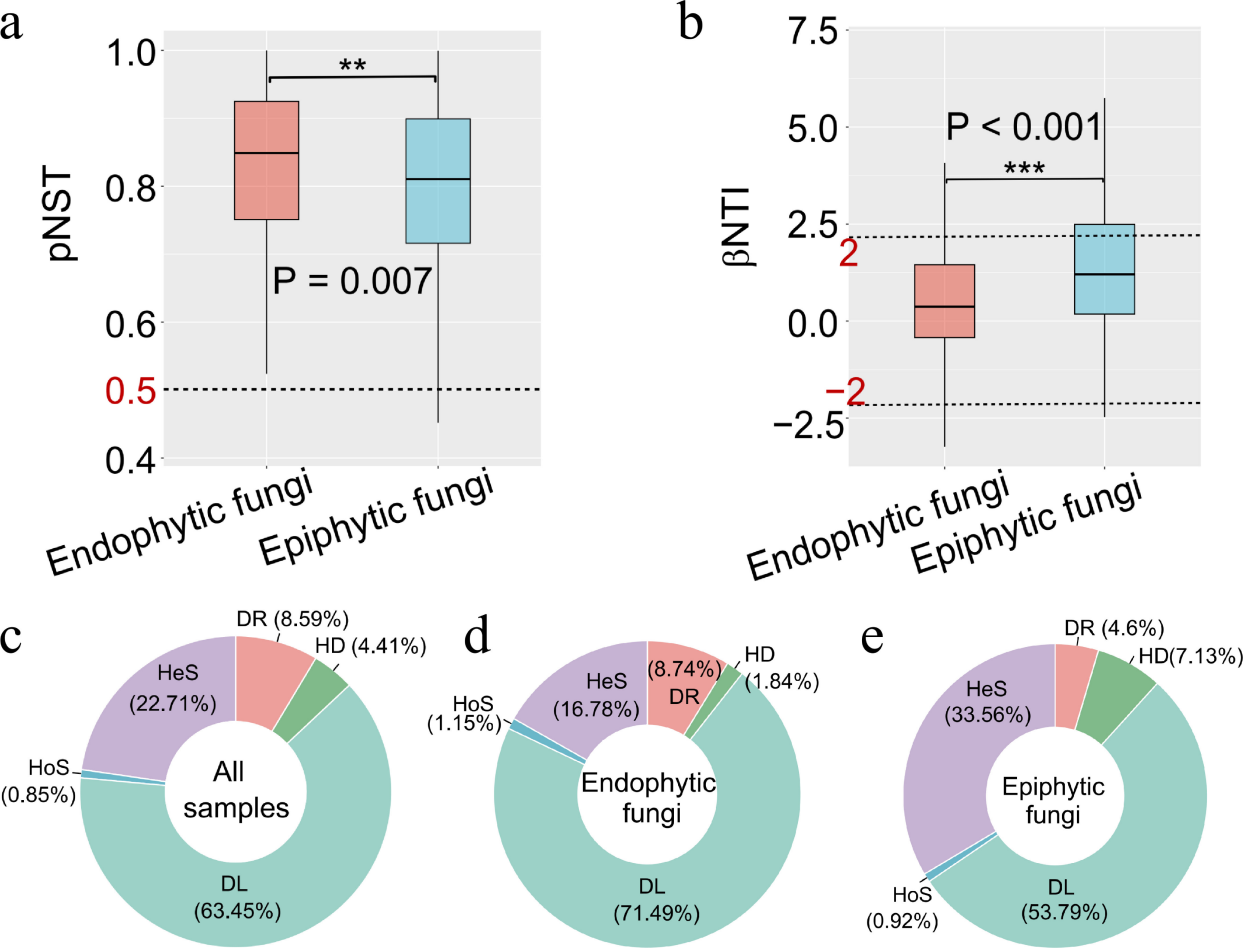
The ecological process of phyllosphere fungal communities. (a) Distribution of fungal community phylogenetic normalized stochasticity ratio (pNST) in all samples based on different ecological niches (endophytic and epiphytic fungi); (b) Distribution of fungal community beta nearest taxon index (βNTI) based on samples of endophytic and epiphytic fungi. The color represents the magnitude of the correlation coefficient. Wilcoxon test was used to test the difference between groups. * indicates significant differences at 0.01 < *P* ≤ 0.05; ** indicates highly significant differences at 0.001 < *P* ≤ 0.01; *** indicates extremely significant differences at *P* ≤ 0.001; (c–e) The assembly process and proportion of all samples, endophytic, and epiphytic fungi. HoS: homogeneous selection; HeS: heterogeneous selection; HD: homogenizing dispersal; DL: dispersal limitation; DR: drift.

We used null model analysis to evaluate the phylogenetically normalized stochastic ratio (pNST) and identified a significant disparity in the pNST ratios between endophytic and epiphytic fungal communities, with both predominantly influenced by stochastic processes (pNST >0.5). Interestingly, the geographic distribution of phyllosphere fungal communities did not alter these ratios (Fig. 5a; Fig. S6). Further investigations with null models, including βNTI and RC_bray_, affirmed the dominant influence of stochastic processes (|βNTI| < 2) on the composition of both community types, while deterministic processes played a relatively minor role (Fig. 5b through e). Notably, RC_bray_ analysis revealed that dispersal limitation was the most significant ecological process, particularly in endophytic fungal communities, where it accounted for 71.49% of the ecological process (Fig. 5d). Beyond dispersal limitation, heterogenous selection also played a significant role in epiphytic fungal communities, accounting for 33.56%, markedly higher than that observed within endophytic fungi (16.78%) (Fig. 5e).

## DISCUSSION

### Composition and diversity of phyllosphere fungal communities

This investigation demonstrates the prevalence of Dothideomycetes and Eurotiomycetes across different sites and ecological niches within phyllosphere fungal communities (Fig. 1a). This pattern is supported by Qian *et al.* (64) concerning the phyllosphere fungal microbiome and is further corroborated by earlier research from Meiser *et al.* (65), who identified a similar dominance of Dothideomycetes, accounting for 21% in phyllosphere fungal communities through meta-analysis. As one of the largest and most ecologically varied groups within the fungal kingdom, Dothideomycetes play a critical role in ecosystem functionality and the global carbon cycle, particularly in the decomposition of cellulose and complex carbohydrates (66). Additional studies by Dissanayake *et al.* (67) and Yao *et al.* (33) have also highlighted the widespread prevalence of these fungal classes in both epiphytic and endophytic communities, underscoring the taxonomic consistency and dominance of these fungi in plant fungal microbiology.

Our observations indicate that geographical regions significantly impact the alpha diversity of both endophytic and epiphytic fungi, with epiphytic fungi demonstrating a notably higher alpha diversity than their endophytic counterparts (Fig. 1b). Through principal coordinates analysis (PCoA) and permutational multivariate analysis of variance (PERMANOVA), our study revealed significant differences in the community structures of endophytic and epiphytic fungi across all samples, with notable regional distribution patterns (Fig. 2). In summary, these differences are likely attributable to variations in altitude, latitude, and environmental conditions at our sampling sites. The observed divergence may stem from the distinct microenvironments of the leaf surfaces inhabited by endophytic and epiphytic fungi, as external environmental factors such as seasonality, rainfall, and elevation shape these fungal communities (11, 68). Additionally, variations in leaf functional traits, such as nutrient content and the physical and chemical properties of the leaves, have been widely reported to influence the composition of phyllosphere fungal communities (69, 70) (Fig. 2). These findings offer valuable insights into the geographical distribution and ecological dynamics of phyllosphere fungal communities.

### Co-occurrence networks of phyllosphere fungal communities

The analysis of co-occurrence networks offers a sophisticated lens through which to view the interactions between plants and their associated fungal communities, advancing beyond traditional assessments of community diversity (71). Our findings demonstrate that epiphytic fungal communities exhibit significantly higher counts of nodes and edges, as well as greater average degree and network density, compared to endophytic communities (Fig. 3a and b; Table S3). Consistent with prior studies analyzing network structures of endophytic and epiphytic fungi in mangroves (72), this indicates that epiphytic fungi participate in more complex and extensive ecological interactions, possibly due to increased niche overlap (73). Additionally, the intricate internal structure of dicotyledonous plants may foster more isolated and dispersed habitats, which can lead to reduced fungal community diversity and fewer interactions among endophytic fungi, thereby simplifying their network structures.

In terms of network topology, pivotal elements like network hubs and connectors play crucial roles in regulating and facilitating connections. Module hubs, although possibly less influential overall, are vital for connecting different network modules and maintaining essential functions (74). Conversely, connectors, which are typically more stable, act as critical links between various groups (75). Identifying such key species is crucial because their removal could lead to network disintegration and loss of stability, fundamental to preserving the integrity of these ecological networks. Targeting these species for microbial management might enhance agricultural productivity (76). Our research identified essential OTUs across all samples, showcasing robust network stability within endophytic and epiphytic fungal communities (Fig. S4).

### Ecological processes governing the composition of phyllosphere fungal communities

The composition of microbial communities is profoundly influenced by ecological processes, such as ecological drift, selection, and dispersal mechanisms (54). A quantitative evaluation of these forces is crucial for understanding ecosystem structure and function in greater depth (58). Utilizing neutral model analysis, our study found that the migration rate (m value) of epiphytic fungi is significantly higher than that of endophytic fungi (Fig. 4), indicating reduced dispersal limitations within epiphytic fungal communities (77). Communities with lower dispersal limitations typically experience strong environmental selection pressures, characterized by the prevalence of a few highly abundant species and greater variability among rarer species (78).

Additionally, this research highlights substantial differences between endophytic and epiphytic fungi in terms of phylogenetically normalized stochastic ratios (pNSTs) and beta nearest taxon index (βNTI) values (Fig. 5b), underscoring the pivotal role of niche differentiation in the composition of phyllosphere fungal communities, a finding in line with Zhong *et al.* (79). Results from null model analyses suggest that the composition of these communities is influenced by both deterministic and stochastic processes, with stochastic processes playing a predominant role. In both types of fungal communities, dispersal limitation was identified as the most significant ecological process (Fig. 5), consistent with results from the neutral model. This emphasis on stochastic processes, particularly dispersal limitation, is supported by previous research which has highlighted its critical role in shaping fungal community composition, reflecting fungi’s strong biogeographical patterns and limited dispersal capabilities (80, 81). On the other hand, the proportion of deterministic processes in epiphytic fungi is significantly higher than in endophytic fungi, primarily driven by heterogeneous selection (Fig. 5d through g). This could be attributed to the heightened responsiveness of epiphytic fungi to external environmental stressors, such as temperature and humidity, compared with endophytic fungi (82). Furthermore, heterogeneous selection refers to the selective pressures exerted under variable biotic and abiotic environmental conditions, resulting in the formation of more divergent community processes (83).

In addition, the dominant stochastic processes observed in phyllosphere fungal community composition can be attributed to a complex interplay of host-specific and environmental factors. Host-related factors, such as genetic diversity, physiological state, and spatial heterogeneity on the leaf surface, introduce variability that allows for random colonization events, leading to community assembly driven by chance rather than deterministic selection (84, 85). Additionally, environmental factors like microclimatic variability, unpredictable dispersal and immigration events, and temporal disturbances further amplify the role of stochasticity (83, 86). The interaction between these host and environmental factors creates a dynamic and heterogeneous environment where fungal community composition is influenced by random fluctuations, making it difficult to predict based solely on deterministic models. Understanding these influences highlights the need for incorporating detailed host and environmental measurements in future studies to better capture the relative contributions of stochastic versus deterministic processes in shaping phyllosphere fungal communities. Future research could benefit from incorporating more detailed measurements of host traits and environmental conditions, as well as examining temporal dynamics to better understand the relative contributions of stochastic versus deterministic processes in shaping phyllosphere fungal communities.

### Conclusions

Our research provides evidence that epiphytic fungal communities display greater diversity than endophytic fungi, with geographic variation significantly impacting the diversity and community composition of both groups. Additionally, co-occurrence analyses indicate that networks of epiphytic fungi exhibit greater complexity, whereas those associated with endophytic fungi demonstrate increased stability. Through neutral and null model evaluations, we discern that the community structure of phyllosphere fungi is regulated by an intricate interplay of deterministic and stochastic processes, with stochasticity predominating. This investigation markedly enhances our comprehension of the ecological determinants shaping phyllosphere fungal communities, providing novel insights into microbial ecology and facilitating pathways for targeted ecological strategies.

## Data Availability

The sequence data were deposited in the National Center for Biotechnology Information (NCBI) Sequence Read Archive (SRA) database (accession no. PRJNA1096448).

## SUPPLEMENTAL MATERIAL

Supplemental material spectrum.01019-24-s0001.docx Fig. S1 to S6; Tables S1 to S3.

ASM does not own the copyrights to Supplemental Material that may be linked to, or accessed through, an article. The authors have granted ASM a non-exclusive, world-wide license to publish the Supplemental Material files. Please contact the corresponding author directly for reuse.
