# Supplementary material for: Comparative analysis of community composition and network structure between phyllosphere endophytic and epiphytic fungal communities of Mussaenda pubescens
Source: Microbiol Spectr. 2024 Dec 3;13(1):e01019-24. doi: 10.1128/spectrum.01019-24 (PMC11705847; doi:10.1128/spectrum.01019-24)
Supplement: Supplemental material — Fig. S1 to S6; Tables S1 to S3. [file spectrum.01019-24-s0001.docx]

**Comparative Analysis of Community Composition and Network Structure between Phyllosphere Endophytic and Epiphytic Fungal Communities of *Mussaenda pubescens***

**Deqiang Chen ^a, b, c^,** **Juanjuan Yang ^c^, Shunfen Wang ^c^, Siren Lan ^a, b, c^, Yonglong Wang ^d *^, Zhong-Jian Liu ^a, b, c *^, Xin Qian ^c *^**

^a^ Fujian Colleges and Universities Engineering Research Institute of Conservation and Utilization of Natural Bioresources, College of Forestry, Fujian Agriculture and Forestry University, Fuzhou 350002, China

^b^ Key Laboratory of National Forestry and Grassland Administration for Orchid Conservation and Utilization at Colleage of Landscape Architecture, Fujian Agriculture and Forestry University, Fuzhou 350002, China

^c^ Fujian Agriculture and Forestry University, Fuzhou 350002, China

^d^ Baotou Teachers’ College, Baotou, 014030, China

^*^ Corresponding author:

Xin Qian, E-mail: qxxb2006@163.com;

Zhong-Jian Liu, E-mail: zjliu@fafu.edu.cn;

Yonglong Wang, E-mail: wylongceltics@163.com


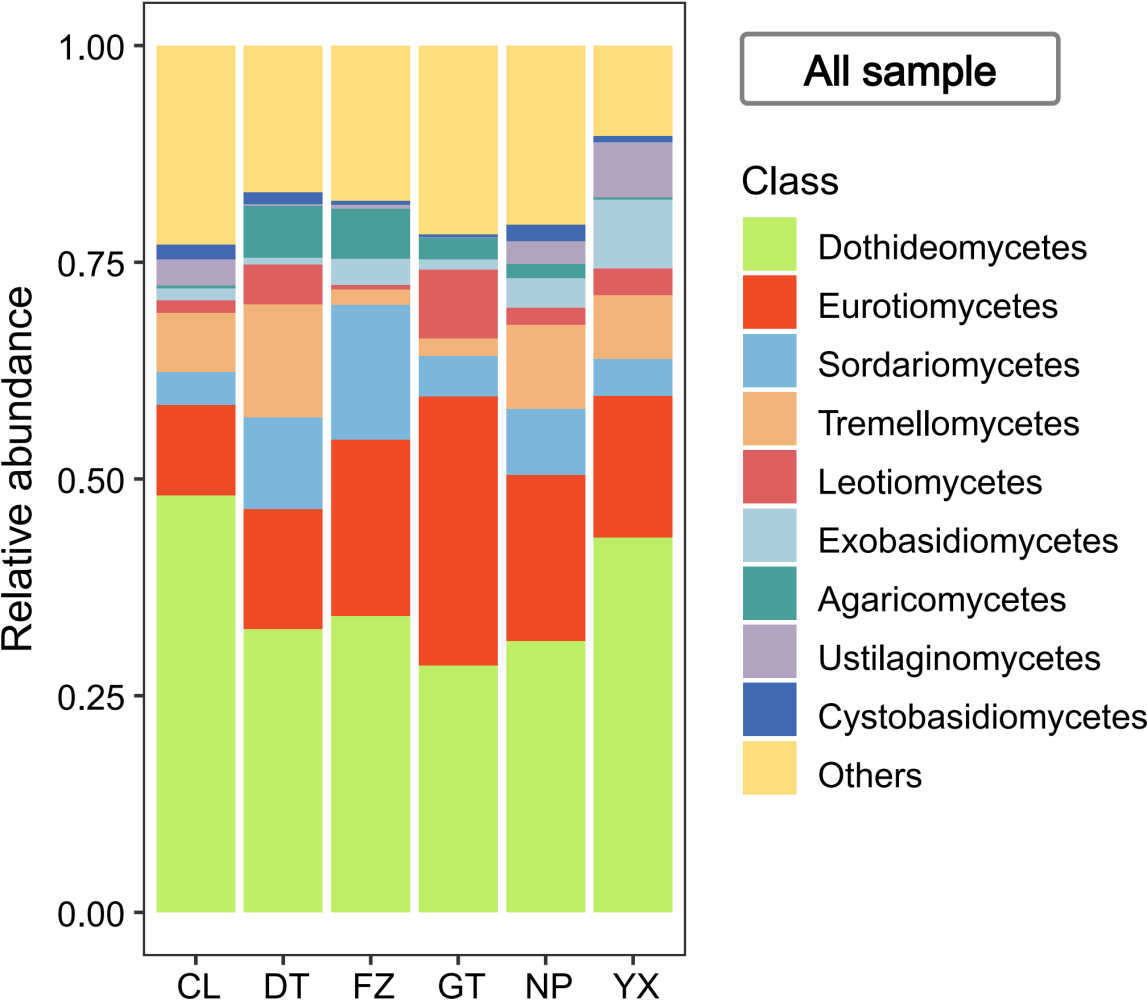


Fig. S1. The relative abundance of the top ten fungal class in all samples based on different geographical locations.


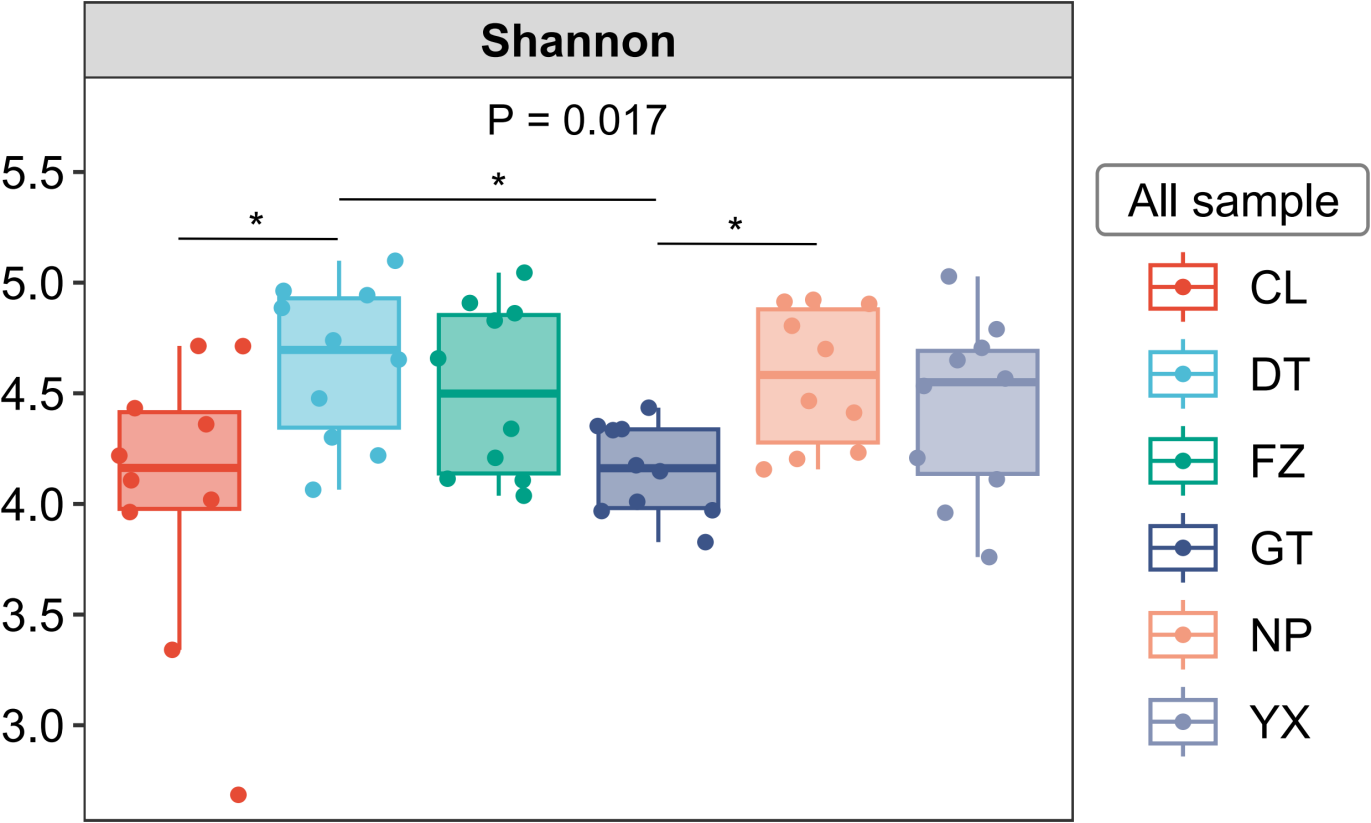


Fig. S2. The alpha diversity (shannon indices) in all samples based on different geographical locations. Kruskal Wallis nonparametric test was used to obtain the *P* value of the difference across all groups, and the Dunn's test is a post-hoc test for differences between each pair of groups. * indicates significant differences at *P* < 0.05; ** indicates highly significant differences at *P* < 0.01; *** indicates extremely significant differences at *P* < 0.001.


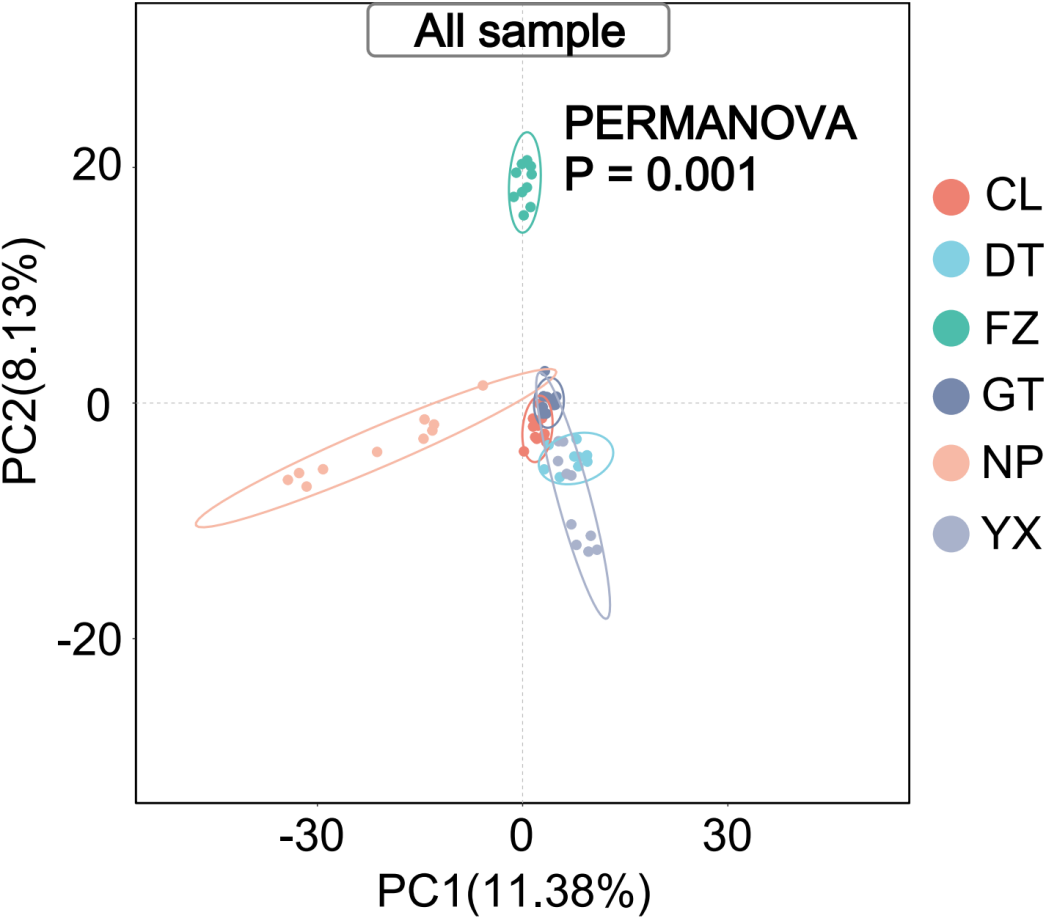


Fig. S3. Principal coordinate analysis (PCoA) and Permutational multivariate analysis of variance (PERMANOVA) of phyllosphere fungal communities across various geographical locations.


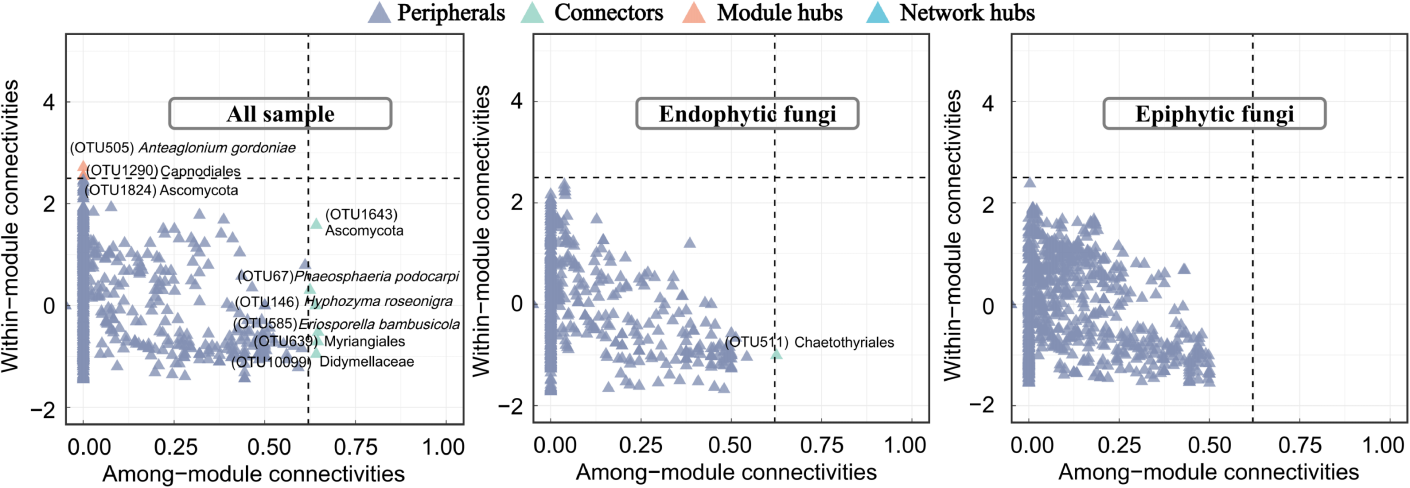


Fig. S4. Zi-Pi plots to detect putative keystone OTUs within the phyllosphere fungal community networks of the all sample, endophytic, and epiphytic sub-communities, respectively. Each point represents an phyllosphere fungal community OTU. The threshold values of Zi and Pi for classification are 2.5 and 0.62, respectively.


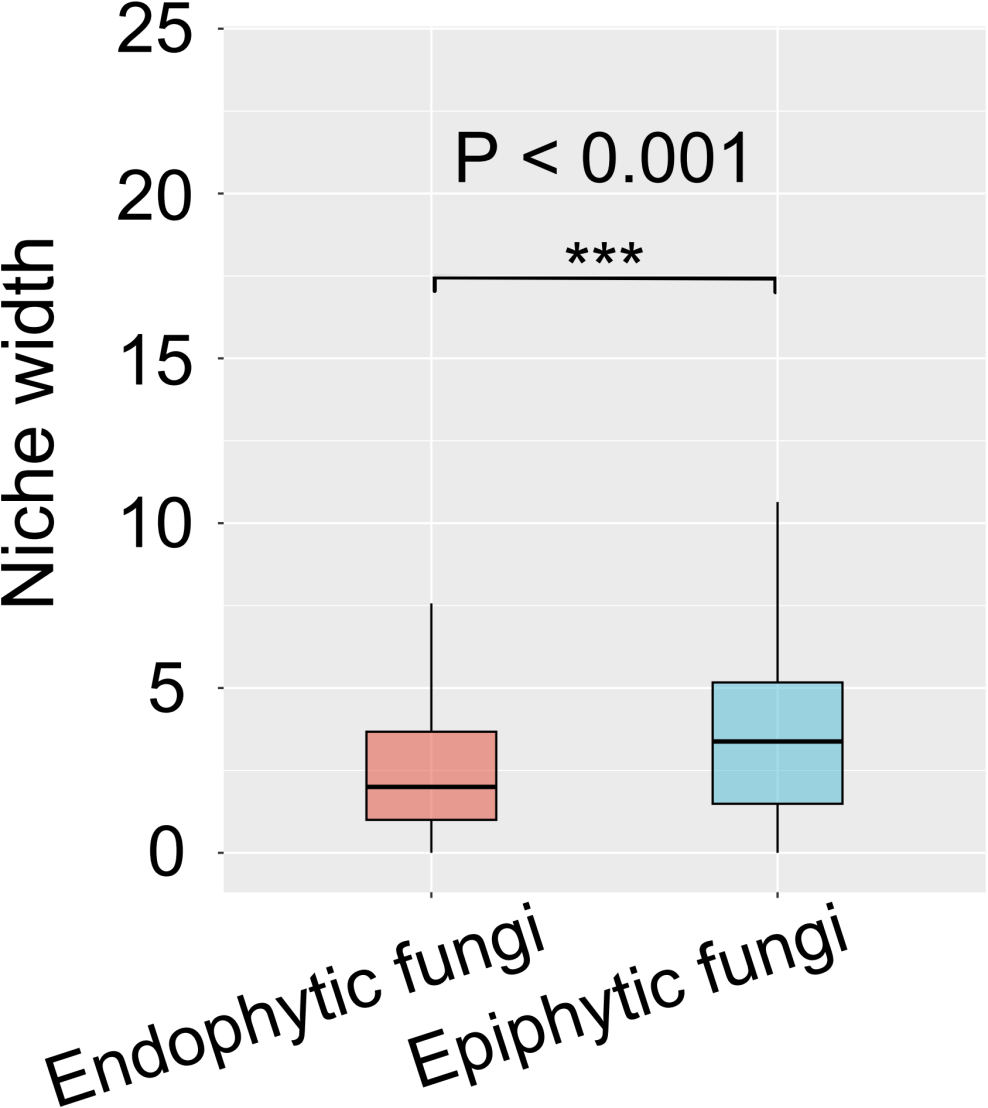


Fig. S5. The niche breadth of the endophytic, and epiphytic fungal communities. Kruskal Wallis nonparametric test was used to obtain the P value of the difference between groups, and Dunn's test was used to test the significance of the difference. * indicates significant differences at P < 0.05; ** indicates highly significant differences at P < 0.01; *** indicates extremely significant differences at P < 0.001.


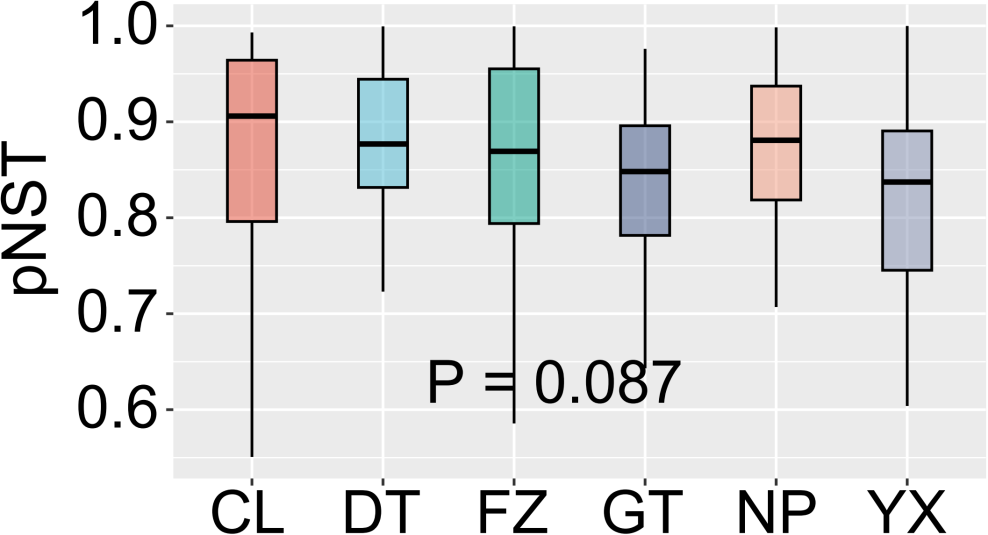


Fig. S6. Distribution of phyllosphere fungal community phylogenetic normalized stochasticity ratio (pNST) in all samples based on different geographical locations.

Table S1. Similarity percentage analysis (SIMPER) of phyllosphere endophytic fungal community structure

| Class | average | sd | ratio | cumsum |
| --- | --- | --- | --- | --- |
| Eurotiomycetes | 0.03772 | 0.024994 | 1.509 | 0.248 |
| Tremellomycetes | 0.0241 | 0.01783 | 1.352 | 0.406 |
| Others | 0.01898 | 0.013224 | 1.435 | 0.531 |
| Exobasidiomycetes | 0.01753 | 0.013951 | 1.257 | 0.646 |
| Dothideomycetes | 0.01541 | 0.011884 | 1.297 | 0.747 |
| Ustilaginomycetes | 0.0114 | 0.008651 | 1.317 | 0.822 |
| Leotiomycetes | 0.01133 | 0.007535 | 1.503 | 0.896 |
| Sordariomycetes | 0.00975 | 0.006516 | 1.496 | 0.96 |
| Agaricomycetes | 0.0034 | 0.003141 | 1.081 | 0.983 |
| Cystobasidiomycetes | 0.00263 | 0.0019 | 1.382 | 1 |

Average: represents the contribution of species to the mean inter-group Bray-Curtis dissimilarity, and the species are sorted by their contribution; SD: standard deviation of the contribution; Ratio: the ratio of Average to SD; Cusum: represents the cumulative contribution of species.

Table S2. Similarity percentage analysis (SIMPER) of phyllosphere epiphytic fungal community structure

| Class | average | sd | ratio | cumsum |
| --- | --- | --- | --- | --- |
| Eurotiomycetes | 0.03453 | 0.023529 | 1.468 | 0.235 |
| Dothideomycetes | 0.03276 | 0.02213 | 1.48 | 0.458 |
| Tremellomycetes | 0.01886 | 0.016917 | 1.115 | 0.586 |
| Agaricomycetes | 0.01378 | 0.009331 | 1.477 | 0.68 |
| Sordariomycetes | 0.01266 | 0.011025 | 1.148 | 0.766 |
| Ustilaginomycetes | 0.01111 | 0.007456 | 1.49 | 0.842 |
| Others | 0.01056 | 0.007039 | 1.5 | 0.914 |
| Exobasidiomycetes | 0.00687 | 0.004966 | 1.384 | 0.961 |
| Cystobasidiomycetes | 0.00335 | 0.00285 | 1.174 | 0.983 |
| Leotiomycetes | 0.00245 | 0.002179 | 1.123 | 1 |

Average: represents the contribution of species to the mean inter-group Bray-Curtis dissimilarity, and the species are sorted by their contribution; SD: standard deviation of the contribution; Ratio: the ratio of Average to SD; Cusum: represents the cumulative contribution of species.

Table S3. Network properties of all sample, endophytic, and epiphytic fungi

|  | Nodes number | Edges number | Average degree | Average path length | Network density | Clustering coefficient | Modularity |
| --- | --- | --- | --- | --- | --- | --- | --- |
| All sample | 917 | 19897 | 43.396 | 2.140 | 0.047 | 0.708 | 0.560 |
| Endophytic fungi | 839 | 17075 | 40.703 | 2.168 | 0.049 | 0.704 | 0.633 |
| Epiphytic fungi | 1106 | 44820 | 81.049 | 1.694 | 0.073 | 0.657 | 0.559 |
